# Development of an App for Symptom Management in Women With Breast Cancer Receiving Maintenance Aromatase Inhibitors: Protocol for a Mixed Methods Feasibility Study

**DOI:** 10.2196/49549

**Published:** 2024-02-15

**Authors:** Trine Lund-Jacobsen, Peter Schwarz, Gabriella Martino, Helle Pappot, Karin Piil

**Affiliations:** 1 Department of Endocrinology, Centre for Cancer and Organ Diseases Copenhagen University Hospital, Rigshospitalet Copenhagen Denmark; 2 Department of Clinical and Experimental Medicine University of Messina Messina Italy; 3 Department of Oncology, Centre for Cancer and Organ Diseases Copenhagen University Hospital, Rigshospitalet Copenhagen Denmark

**Keywords:** acceptability, aromatase inhibitors, breast cancer, cancer, chemotherapy, disease, feasibility, inhibitor, management, mHealth, postmenopausal, psychosocial, QoL, quality of life, radiation therapy, symptom management, symptom, tool, treatment, usability, user-friendliness

## Abstract

**Background:**

Patients with postmenopausal nonmetastatic estrogen receptor–positive breast cancer often experience a reduced quality of life after primary treatment. The disease and treatment trajectory consists of surgery followed by chemotherapy or radiation therapy. Upon this, maintenance hormone therapy with an aromatase inhibitor can result in several physical and psychosocial symptoms. Optimal symptom control during maintenance therapy is central to maintaining the patient’s quality of life.

**Objective:**

This study aims to (1) develop an electronic symptom management tool for patients with postmenopausal early breast cancer receiving maintenance aromatase inhibitors with an endocrine aspect and (2) assess the feasibility, acceptability, and usability of the pilot version of the Bone@BC app. Furthermore, longitudinally, symptom prevalence and quality of life for patients with postmenopausal nonmetastatic estrogen receptor–positive breast cancer will be explored.

**Methods:**

This study follows a multistage research plan. In stage 1, a systematic literature review to establish an overview of aromatase inhibitor–related symptoms reported by postmenopausal women with nonmetastatic estrogen receptor–positive breast cancer will be completed. In stage 2, a comprehensive overview of symptoms related to aromatase inhibitors (letrozole, exemestane, and anastrozole) will be performed (eg, by reviewing medical leaflets and guidelines). In stage 3, an electronic app with a user-friendly Patient Concern Inventory list to comprise symptoms and concerns will be developed. Last, in stage 4, a convergent mixed methods feasibility study of the pilot version of the Bone@BC app will be conducted. A total of 45 patients with postmenopausal nonmetastatic estrogen receptor–positive breast cancer will use the app daily for symptom identification and respond to 6 serial patient-reported outcome measurements for 12 weeks. Finally, semistructured interviews will be performed. The primary outcome includes consent rate, attrition rate, retention rates, technical issues, and adherence, assessed using preestablished criteria on feasibility and a mixed methods approach for exploring acceptability. A patient advisory board consisting of 5 women with breast cancer is recruited to include their perspectives and experiences in the planning, organization, implementation, and dissemination of the research throughout the project.

**Results:**

At the time of submitting this paper (January 2024), a total of 23 patients have been included in the stage 2 medical audit over the recruitment period of 3 months (November 2022 to February 2023), and 19 patients have been enrolled in stage 2, the semistructured patient interviews.

**Conclusions:**

This protocol describes a study investigating the feasibility, acceptability, and usability of the symptom management tool Bone@BC developed for patients with breast cancer with an endocrine aspect.

**Trial Registration:**

ClinicalTrails.gov NCT05367830; https://clinicaltrials.gov/ct2/show/NCT05367830

**International Registered Report Identifier (IRRID):**

DERR1-10.2196/49549

## Introduction

### Breast Cancer

Patients with postmenopausal nonmetastatic estrogen receptor–positive (ER+) breast cancer (BC) in maintenance therapy with an aromatase inhibitor (AI) deal with numerous long-term cancer- and treatment-related side effects (eg, affected bone health). These side effects lead to impaired health-related quality of life (HRQoL) even years after ending primary treatment and during maintenance hormone therapy [1-3].

### The Transition

The transition from being closely monitored by specialists to fewer follow-up visits is difficult for the survivors of BC [4]. These difficulties are explained by reduced interaction with and less psychological support from health care professionals (HCPs) [5-8], as well as, in light of the less frequent consultations, the fact that their surroundings begin to consider the survivors of BC to be cured and healthy [9]. Still, patients during this trajectory stage must be capable of reacting sufficiently to the experienced side effects and potential symptoms [9]. Hence, understanding where and how information and support can be sought for these women is crucial [10] to optimize their self-efficacy and self-management [11].

### Patient-Reported Outcomes and Mobile Health

Patient-reported outcomes (PROs) are an important element in the person-centered care of patients with cancer [12,13]. Numerous applications have been developed to collect electronic patient-reported outcomes (ePROs) [14-16]. Mobile health (mHealth) apps have the potential to increase patients’ self-efficacy, strengthen empowerment, and offer value to patients in their daily lives [17,18]. The growing field of mHealth has been applied to numerous areas, including health promotion, behavior change support, and self-management of cancer diseases. mHealth is a subset of digital health, or eHealth, which also includes health information, telemedicine, and personalized medicine [19]. mHealth can be quickly scaled to reach thousands of people and potentially increase access to health care. mHealth is well-suited to symptom management, as it can provide timely dissemination of health information, encourage patients to acquire information during their communication with clinicians, and guide self-management.

### Hypothesis

We hypothesize that patients with postmenopausal nonmetastatic ER+ BC often experience reduced HRQoL after primary treatment due to treatment-related symptoms. Furthermore, transitioning from being closely monitored by HCPs to fewer follow-up visits can indeed be challenging for some patients. The close monitoring provided by HCPs can provide a sense of security and reassurance, and reducing the frequency of visits may therefore lead to feelings of uncertainty or anxiety. To help patients navigate this transition more smoothly, we will develop a symptom management tool in the form of a pilot version of the Bone@BC app. The pilot version of the Bone@BC app has the potential to significantly support patients in several ways, leading to an improved HRQoL and enhanced communication with HCPs. The app can include features that enable patients to monitor their symptoms regularly so that they will be able to act on them if necessary. It is important to note that the effectiveness of the Bone@BC app will depend on its design, functionality, and user experience. Conducting user testing and gathering feedback during the pilot phase will help identify areas for improvement and ensure that the app meets the specific needs of the target group of patients.

## Methods

### Study Design

**Figure 1:**
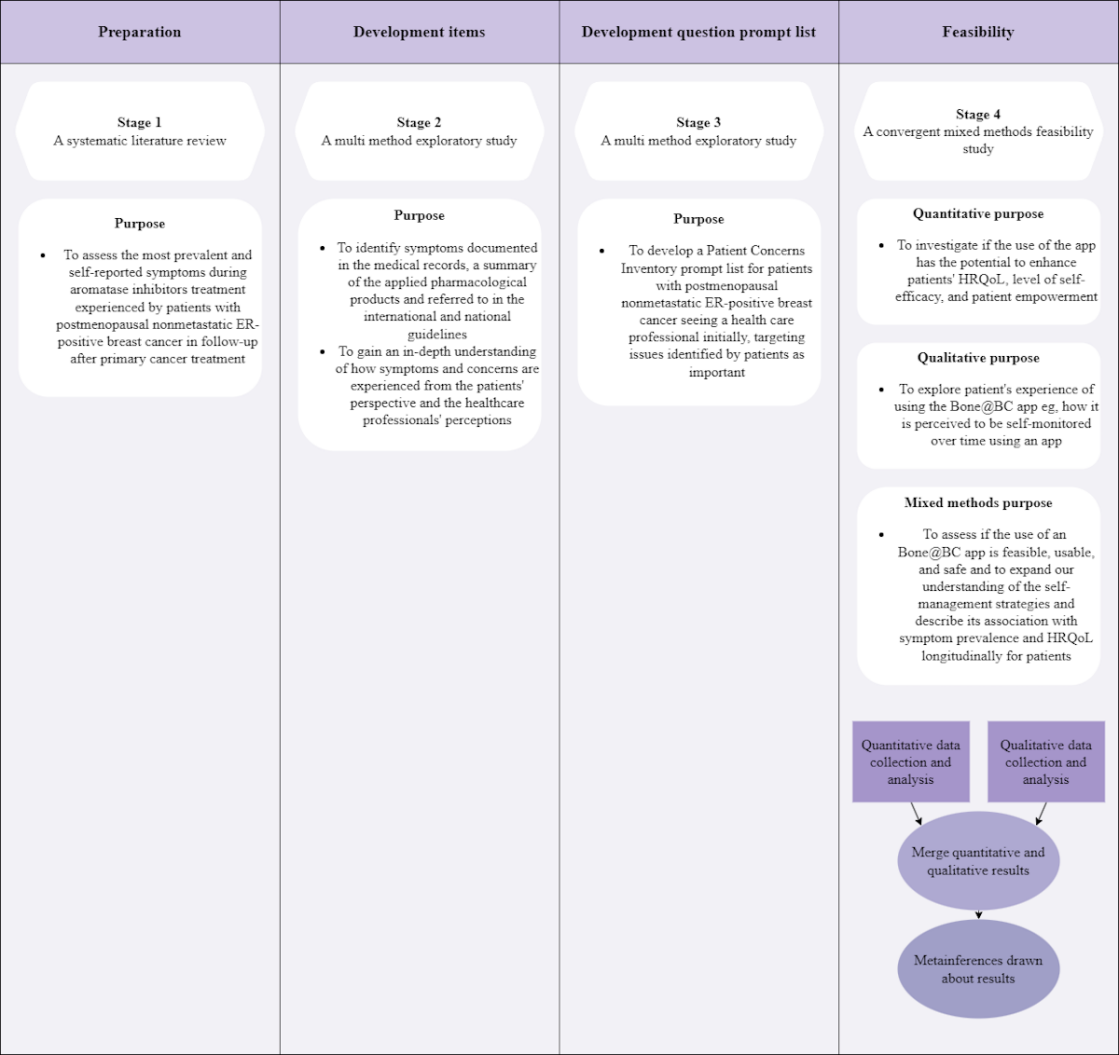
A multistage feasibility study. Convergent refers to the quantitative and qualitative data set being collected concurrently. Merge mean data will be analyzed separately and then merged according to allowing the research purpose from multiple perspectives. Metainferences refer to quantitative and qualitative results combined in a conclusion. ER: estrogen receptor; HRQoL: health-related quality of life.

This study follows a multistage feasibility design [20,21] comprising 4 stages (Figure 1 [21]).

As recommended in *Guidelines for Reporting Non-Randomized Pilot and Feasibility Studies* by Lancaster and Thabane [22], the reporting of the study protocol adheres to the CONSORT (Consolidated Standards of Reporting Trials) extension to pilot and feasibility trials.

### Study Setting

This study will be carried out as a single-center study at the endocrinology outpatient clinic at the Copenhagen University Hospital, Rigshospitalet, in Denmark.

### Recruitment

The patients will be recruited from the endocrinology outpatient clinic by clinicians at routine consultations. The semistructured interviews will be performed by the principal investigator (TLJ) not being involved in the treatment and care of these patients. Patients will be screened for inclusion criteria during routine appointments. The informed consent can be withdrawn at any time. A screening log will be kept collecting reasons for nonresponses.

### Eligibility

The predetermined eligibility criteria are women aged between 50 and 70 years with a diagnosis of postmenopausal nonmetastatic ER+ BC; in maintenance therapy with an AI (letrozole, anastrozole, or exemestane); who are able to understand, read, and speak Danish; have access to a smartphone that can display the app (eg, iOS, iPADOS, or Android); and provide informed consent. Patients will be considered ineligible if they are unable to provide informed consent due to cognitive or linguistic inability, have a physiological or cognitive impairment that would prevent or inhibit the participation in using the app and answering the ePROs, have a previous malignancy, or are in maintenance therapy with tamoxifen.

### Patient and Public Involvement

Because patient involvement is essential for this study, to ensure patient-centeredness [23], a patient advisory board consisting of 5 women diagnosed with BC was recruited through an advertisement on the Danish Breast Cancer Organization’s website. The primary objective of the patient advisory board is to enhance the researcher’s communication and cooperation with patients, thereby ensuring the integration of their perspectives, requirements, experiences, and expectations throughout the various stages of research, including planning, organization, implementation, and dissemination [24,25].

### Intervention Description

#### The Proof-of-Concept Version of the Bone@BC App

**Figure 2:**
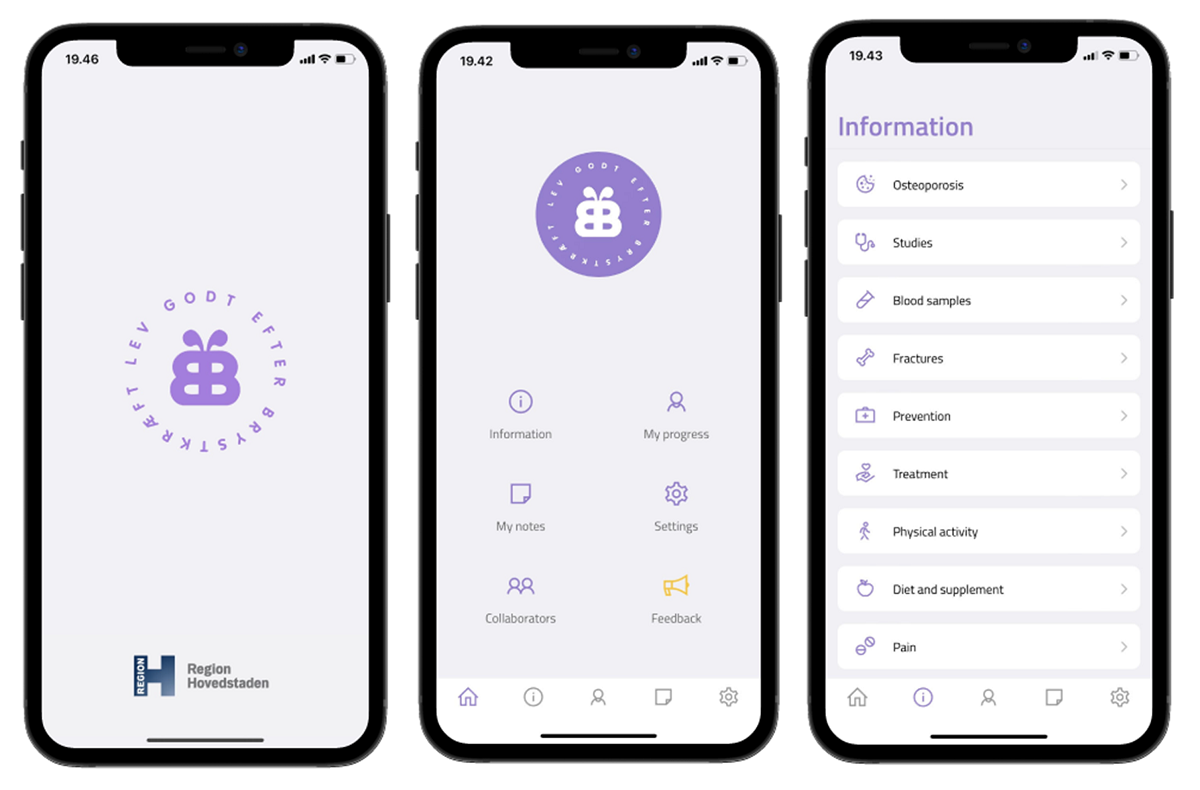
Overview of the proof-of-concept version of the Bone@BC app.

Originally, a proof of concept of the Bone@BC app was developed (2015-2018) by a team of clinicians with specialist experience from the Healthy Living After Breast Cancer research group. The proof-of-concept app was published in 2 languages, Danish and English. The proof-of-concept version provides (1) advice on treatment elements (eg, blood samples and prevention); (2) daily questions about daily living; and (3) private notes. However, the proof-of-concept version of the app did not follow a systematic selection of symptoms to include, and no patients were involved in the design or content of the app (Figure 2).

#### The Pilot Version of the Bone@BC App

The proof-of-concept version of the app yielded several noteworthy patient feedback examples, which are as follows:

- Patients expressed the desire for an app that goes beyond educational purposes and provides support in their daily lives. Specifically, they highlighted the importance of having a tool that helps them remember changes in their symptoms.
- Patients emphasized the need for visual information, such as trends indicating their quality of life based on daily responses to HRQoL assessments.
- Patients expressed the expectation that the app should assist them in identifying relevant topics to discuss with HCPs.
- Some patients did not find the notes module in the app useful.
- Additionally, certain features such as blood samples, daily habits, and dual-energy x-ray absorptiometry scan were deemed unhelpful by patients.

This study seeks to develop a pilot version of the Bone@BC app based on the above feedback from the patients who used the proof-of-concept version of the Bone@BC app. The pilot version of the Bone@BC app will increase patient friendliness by implementing functionalities developed systematically in collaboration with patients and clinicians to solve patients’ specific needs using this symptom management tool (Table 1).

**Table 1:** Overview of components in the proof-of-concept version versus the pilot version.

Components	Proof-of-concept version	Pilot version
Information on treatment-related osteoporosis, blood samples, and physical activity (both text and video) with health care professionals	✓	✓
Training instructions videos		✓
My progress	✓	
My results (eg, blood samples)	✓	
My medication (eg, breast cancer medication)	✓	
Body measures (height and weight)	✓	
Daily habits	✓	
Activity from your mobile phone health tracker		✓
My notes	✓	
Feedback	✓	✓
Patient Concerns Inventory prompt list		✓
Reminder module		✓
Reminder list based on the previous Patient Concerns Inventory prompt list		✓
Intelligent progress of the health-related quality of life measurements		✓
Trends for the health-related quality of life measurements (tendencies algorithm)		✓
Simplified user interface		✓
Danish version	✓	✓
English version	✓	✓

### Definition of Symptoms

Our study will use the definition of symptoms from the National Institutes of Health and the National Cancer Institute: “A physical or mental problem that a person experiences that may indicate a disease or condition. Symptoms cannot be seen and do not show up on medical tests. Some examples of symptoms are headache, fatigue, nausea, and pain” [26].

### Development of the Pilot Version of the Bone@BC App

To develop the pilot app version, this multistage study consists of 4 stages (Figure 1) where the Bone@BC app will be further developed and then tested in a feasibility study.

#### Stage 1

A systematic review will be undertaken to appraise the current literature and provide an overview of AI-related symptoms reported by postmenopausal women with nonmetastatic ER+ BC. The systematic literature review will be reported according to the PRISMA-P (Preferred Reporting Items for Systematic Review and Meta-Analysis for Protocols) 2015 statement [27]. A comprehensive search will be undertaken on the following databases: PubMed, MEDLINE, CINAHL, Embase, Cochrane, Web of Science, PsycINFO, and Scopus. The Mixed Methods Appraisal Tool version 2018 [28] will be used for assessing the included studies’ methodological quality.

#### Stage 2

Stage 2 will be an explorative multimethod stage relying on patient involvement. Moreover, symptoms and concerns identified by the European Organization for Research and Treatment of Cancer (EORTC) Library Item [29] will be selected to be included in the Bone@BC app.

**Figure 3:**
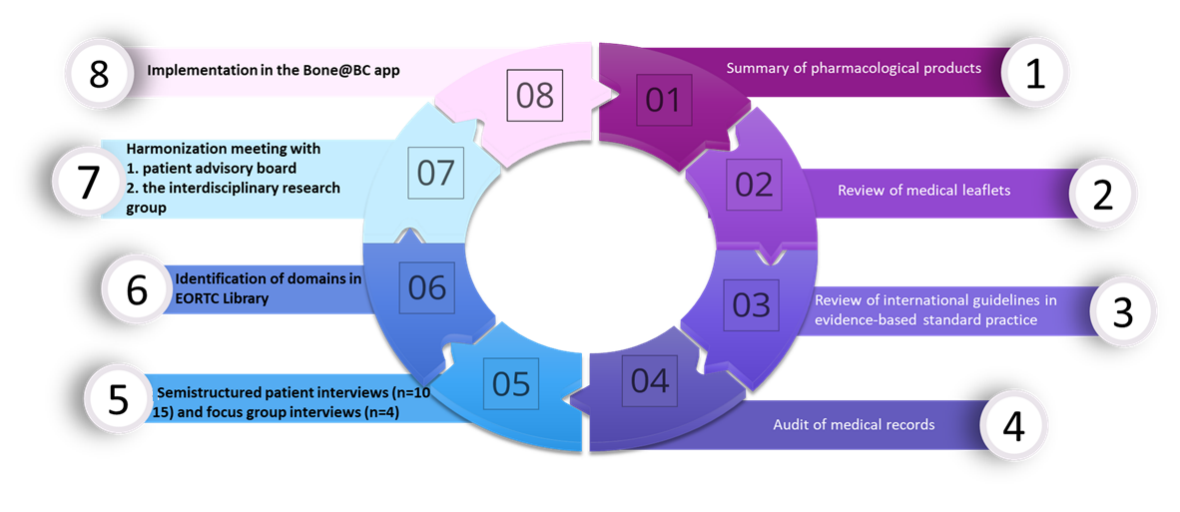
Study assessments in stage 2. EORTC: European Organization for Research and Treatment of Cancer.

Multimethod data collection will be carried out to explore the symptoms reported by the patients with postmenopausal nonmetastatic ER+ BC in maintenance therapy with AI (letrozole, anastrozole, and exemestane; Figure 3).

1. A summary of pharmacological products at the European Medicines Agency [30] and the Food and Drug Administration [31].
2. Review of medical leaflets at the website Medicine.dk [32].
3. Reviews of international and national guidelines for the standard clinical practice: oncological (American Society of Clinical Oncology [33], European Society for Medical Oncology [34], and Danish Breast Cancer Group [35]) and endocrinological (Endocrine Society [36], European Society of Endocrinology [37], and Danish Endocrinological Society [38]).
4. Medical record audit from patients with postmenopausal nonmetastatic ER+ BC. The inclusion period will be from November 14, 2022, to February 14, 2023. All patients attending routine consultations will be invited to participate. The principal investigator will perform the reviews and exclude patients who do not meet the predetermined inclusion criteria.
5. Semistructured face-to-face patient interviews on symptom experience (n=15-20).
6. The quantitative and qualitative results from steps 1 to 5 will, together with the systematic literature review, provide a comprehensive overview of the symptoms, side effects, and concerns that patients with postmenopausal early BC are dealing with. These symptoms and concerns will be organized into domains for HRQoL measurements to identify related and validated items in the EORTC Library that can be implemented in the pilot version of the Bone@BC app daily questions [29].
7. Finally, a harmonization meeting will be organized, initially involving the patient advisory board, followed by the interdisciplinary research group (the authors). The purpose of this meeting will be to facilitate discussion, collaboration, and consensus among participants regarding the selection of specific items from the EORTC Library [39].

#### Stage 3: Multimethod Exploratory Study

In this stage, an electronic Patient Concerns Inventory list (PCI) will be adapted from the English version of the PCI developed by Kanatas et al [40] for patients with BC. The adapted PCI will be modified to fit the app. A PCI is a structured list of frequently asked questions and concerns. It is designed to support and encourage patients to acquire information during their communication with HCPs. The development of the electronic PCI relies on the following steps: (1) translation and linguistic validation according to the Professional Society for Health Economics and Outcomes Research [41]; (2) focus group interview with the patient advisory board; (3) development of a minimum variable product (MVP) app only to test the electronic PCI list; (4) the patient advisory board testing the MVP app and evaluating it in a focus group interview; (5) pilot-test patients (n=15) using the MVP app will be interviewed by semistructured face-to-face interviews; and (6) harmonization meeting in the multidisciplinary research team.

The identified items from the EORTC Library [39] and the PCI will be implemented in the pilot version of the Bone@BC app.

#### Stage 4

**Figure 4:**
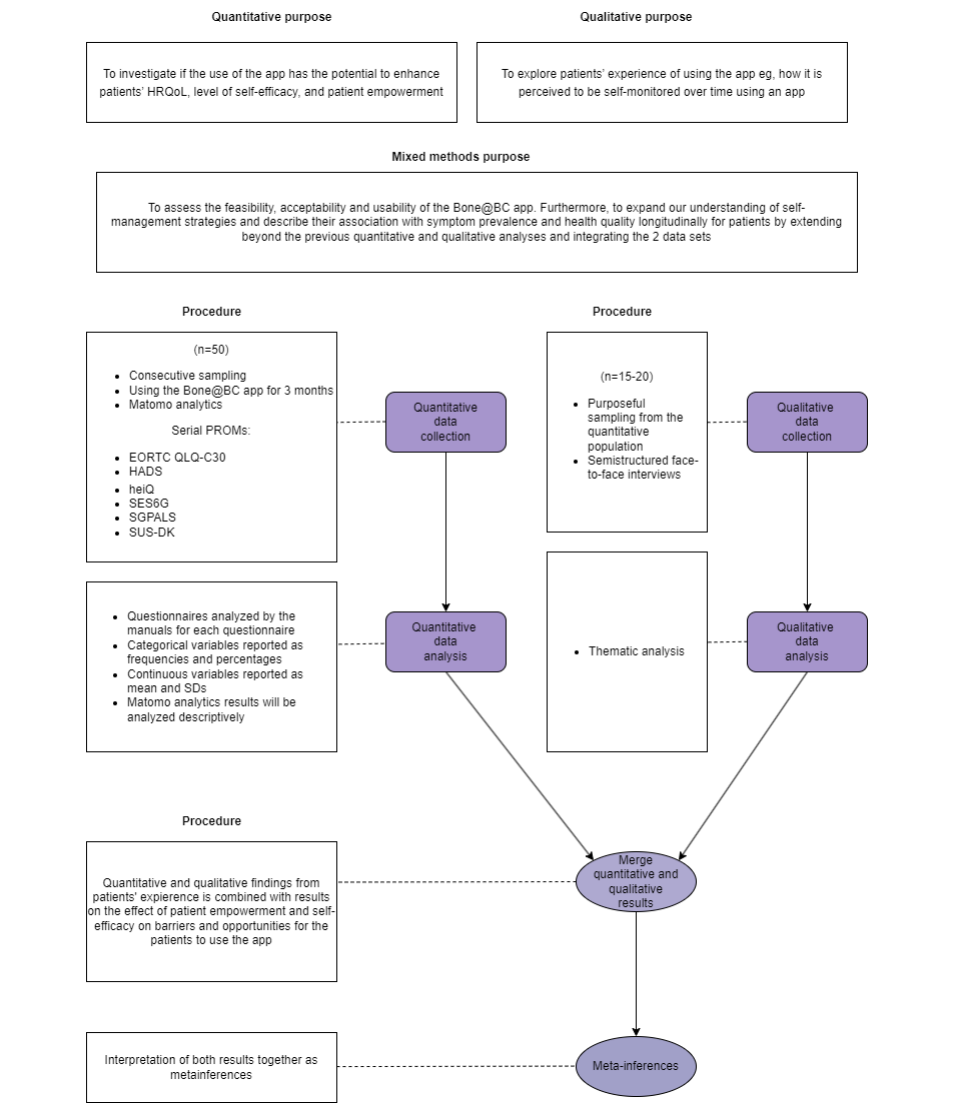
Stage 4: convergent mixed methods feasibility study. Convergent mean that the quantitative and qualitative data sets are collected concurrently, analyzed separately, and then merged accordingly to achieve the research purpose from multiple perspectives. After analysis of each data set, inferences will be drawn. At the end of the study, the metainferences will be drawn and included in the larger interpretation being made in the study’s discussion section. Consecutive sampling refers to every participant who meets the criteria of inclusion and is selected until the required sample size is attained [50]. Purposeful sampling refers to the researcher intentionally recruiting participants who have experienced the central phenomenon being explored in the study. Matomo refers to an open-source digital analytics platform (Matomo). EORTC QLQ-C30: European Organization for Research and Treatment of Cancer Quality of Life C30; HADSA: Hospital Anxiety and Depression Scale; heiQ: Health Education Impact Questionnaire; HRQoL: health-related quality of life; PROM: patient-reported outcome measure; SES6G: Self-Efficacy for Managing Chronic Disease 6-Item Scale; SGPALS: Saltin-Grimby Physical Activity Level Scale; SUS-DK: System Usability Scale–Danish version.

Stage 4 is a convergent, nonrandomized, single-arm mixed methods feasibility study [21] (Figure 4 [20,21,42-49]) investigating the feasibility of the pilot version of the Bone@BC app.

The purpose of this stage will be to explore the patients’ perspectives on feasibility, acceptability, and usability while being offered the pilot version of the Bone@BC app, which will be provided to them as a tool for symptom identification over a period of 12 weeks. Moreover, the consent rate, attrition rate, adherence rate, and retention rate will be explored. Furthermore, the potential changes in self-efficacy, HRQoL, and patient empowerment over time will be measured. The findings from the quantitative data (PRO questionnaires and data in the Bone@BC app) and the qualitative data (patient interviews) will be compared and merged. The intent is to obtain a more comprehensive understanding of the feasibility, usability, barriers, and subgroup of patients who may benefit from using the app.

### Measures

#### Feasibility

The feasibility of the study will be assessed by the rate of recruitment and retention over the study duration (12 weeks). The feasibility parameters include adherence rate, acceptability, response rate, representativeness, recruitment rate, technical difficulties, and satisfaction [51].

#### Acceptability

The acceptability of the pilot version of the Bone@BC app will also be explored by semistructured interviews with a subgroup of the 40-50 invited participants in the study.

#### Usability

The satisfaction survey will be the System Usability Scale–Danish version (SUS-DK) [42] and will be explored in semistructured patient interviews after the intervention. The System Usability Scale is a 10-item questionnaire with 5 responses ranging from “strongly disagree” to “strongly agree” [42]. The final question allows respondents to provide further comments in an open-ended format. The criteria for being satisfied will be a system usability score of ≥68% [42].

#### App Use

The open-source web analytic platform Matomo Analytics [43] will be used to investigate user statistics and traffic in the app. In-app user analytics will be collected to track user behavior such as the number of app sessions, length of app sessions, frequency of use, date the app was first opened, the number of pages, time spent on the pages, and bounce rate.

#### Health-Related Outcomes

Health-related outcomes will be collected at baseline upon registration for the pilot version of the Bone@BC app and then at 3 different time points (Table 2).

**Table 2:** Study assessments and time points stage 4.

Data Collection (n=50)			Baseline		Week																					
					1		2	3		4 (~1 mo)		5		6		7		8 (~2 mo)		9		10		11		12 (~3 mo)
**Data for the research database REDCap^a^**																										
	Informed consent	✓																								
	Demographic data^b^	✓																								
	EORTC QLQ-C30^c^	✓							✓																✓	
	SES6G^d^	✓							✓								✓								✓	
	heiQ^e^	✓																							✓	
	HADS^f^	✓																							✓	
	SGPALS^g^	✓							✓								✓								✓	
	SUS-DK^h^																								✓	
	Open-source web analytic platform Matomo Analytics 3.0^i^	✓							✓								✓								✓	
**PRO^j^ data through the app**																										
	Electronic patient-reported PRO questionnaires on symptoms Bone@BC (everyday)^k^	✓		✓		✓		✓	✓		✓		✓		✓		✓		✓		✓		✓		✓	
Semistructured interviews																										✓

^a^REDCap: Research Electronic Data Capture.

^b^Demographic data (eg, marital status, family status, educational level, and occupation).

^c^EORTC QLQ-C30: European Organization for Research and Treatment of Cancer Quality of Life C30.

^d^SES6G: Self-Efficacy for Managing Chronic Disease 6-Item Scale.

^e^heiQ: Health Education Impact Questionnaire.

^f^HADS: Hospital Anxiety and Depression Scale.

^g^SGPALS: Saltin-Grimby Physical Activity Level Scale.

^h^SUS-DK: System Usability Scale–Danish version.

^i^Open-source web analytic platform Matomo Analytics.

^j^PRO: patient-reported outcome.

^k^Patient-reported outcomes from the app on the questions provided daily on health-related quality of life, late side-effects, symptoms and concerns perspectives, and level of physical activity in the Bone@BC app and question prompt list. Items developed in stage 3 and implemented from the European Organization for Research and Treatment of Quality of Life Item Library in the domains of late side-effects and symptoms management.

The health-related ePROs being assessed before and after the study will be the HRQoL by the 30-item EORTC Quality of Life C30 (QLQ-C30) [44]. The HRQoL domains are divided into multi-item subscales: functional (physical, role, cognitive, emotional, and social), symptom (fatigue, pain, nausea/vomiting, and dyspnea), financial adversity, and global health status [44]. The EORTC-QLQ-C30 has proven to be reliable and valid in a range of patient populations and a variety of treatment settings [52].

Self-efficacy will be measured by the Self-Efficacy for Managing Chronic Disease 6-Item Scale (SES6G) [45]. SES6G is a self-administered questionnaire with 6 items on the patient’s perceived self-efficacy on a 10-point Likert scale ranging from “not at all confident” to “totally confident” [45]. Ritter and Lorig [53] conducted 2 new studies and reviewed 8 independent studies to investigate the psychometric properties of the scale. Cronbach α was a minimum of .88 across all studies; minimal floor and ceiling effects were observed; the measure was sensitive to change; and moderate and significant correlations provide convergent validity evidence when measured against selected health indicators [53].

Physical activity will be measured by the Saltin-Grimby Physical Activity Level Scale (SGPALS) [46]. SGPALS is a 4-level scale. The questionnaire measures the level of physical activity as 3 months before the patients were diagnosed with BC and how the level of physical activity is today [46]. The SGPALS is found to be reliable, with a high level of validity and consistency [54].

Anxiety and depressive symptoms will be measured by the Hospital Anxiety and Depression Scale (HADS) [47]. HADS is a validated screening tool and includes 14 questions addressing anxiety and depressive symptoms with 7 items each in the previous 7 days [47]. In an updated literature review, the HADS was found to be reliable with a Cronbach α between .70 and .90 [55].

Patient empowerment will be measured by the Health Education Impact Questionnaire (heiQ) [48]. heiQ is an outcome and evaluation measure for patient education and self-management interventions for people with chronic conditions. heiQ is a validated screening tool and includes 42 questions with the following 4 options for the answer: “strongly disagree,” “disagree, agree,” or “completely agree” [48]. The heiQ is a valid, reliable measure of key dimensions of generic health-related empowerment [56].

### Statistical Analysis

#### Preestablished Criteria

Feasibility will be explored by looking at the success threshold, attrition rate, and adherence. The success threshold of ≥60% will be defined as the proportion of informed patients giving consent. The attrition rate will be calculated as the proportion of participants withdrawing from the intervention, leaving no data on outcomes available. The retention rate of ≥85% will be the number of individuals who remained in the study and responded to the daily PRO in 12 weeks. The retention rate and success threshold are based on a recent systematic review of internet-based supportive care for patients with lung disease [57]. Patient adherence will be the proportion of patients completing self-reports for each time point adjusted for withdrawals. The adherence rate is the proportion of patients replying to ≥80% of the daily PRO questions. Adherence to daily completion will be analyzed according to, for example, material status and educational level using the Fisher exact test.

Acceptability will be assessed based on the following predetermined criteria: (1) system usability score ≥68% [42]; (2) patients’ experience identified in follow-up interviews; and (3) HRQoL must be at least at the same level before and after the intervention as measured by the EORTC QLQ-C30 [44].

#### Quantitative Data

The quantitative data will be exported to R software (version 4.1.2; R Foundation for Statistical Computing) [58]. All questionnaires will be scored according to the specific manuals. The analysis of the obtained data will be based on CIs and will focus on exploring the longitudinal changes over time. The daily self-report of symptoms will be analyzed using multiple linear regression after the variables have been checked by diagnostic plots to see if they meet the following 5 main assumptions: (1) linearity, (2) homoskedasticity, (3) independence of errors, (4) normality, and (5) independence of independent variables. Descriptive statistics will be performed to describe the sociographic and clinical characteristics. The categorical variables will be reported as frequencies and percentages. Continuous variables will be tested for normality before we decide to report them as mean and SD or median and IQR. In addition, an open-source digital analysis platform (Matomo) [43] will be used to track traffic and user behavior on the app.

#### Qualitative Data

The qualitative data will be collected through individual interviews. All interviews will be recorded and transcribed verbatim. The transcripts will be handled systematically in NVivo (QSR International Pty Ltd) [59] to create an audit trail and facilitate transparency [60]. The observational data and interviews will be analyzed based on the 6 steps of thematic analysis outlined by Braun and Clarke [61,62]. Using inductive coding, transcripts will be interpreted, and themes will be generated. Furthermore, researcher triangulation will strengthen the credibility of the results [60].

### Power

For the semistructured patient interviews, the sample size will be guided by the notion of information power, according to Malterud et al [50]. Being a feasibility study with predetermined criteria for success, a formal sample size calculation is not necessary [63]. However, sample sizes of 40-50 participants have been recommended for feasibility studies [64]. Thus, 40-50 participants will be recruited for the intervention.

### Ethical Considerations

The research will be carried out following the Declaration of Helsinki [65], the General Data Protection Regulation [66], and the Human Research Ethics Committee Denmark [67]. Ethical approval has been obtained from the ethical committee of the Capital Region of Denmark (jr nr 210777457). Data are reviewed and registered in the Capital Region of Denmark (Pactius jr nr P-2022-162). All participants will be required to fill out an informed consent after verbal and written information about the study have been given. A screening logbook will be performed.

The systematic literature review is registered in PROSPERO (ID: DR42021281012). The Bone@BC app is data-reviewed and registered in the Capital Region of Denmark (jr nr 6203, local jr nr RH-2018-38, Pactius jr nr P-2020-520). The Bone@BC app has been approved for the integration of the entered app data with region security requirements and is an official Region Capital of Denmark app. The app uses MITID log-in (national electronic personal ID) and a disclaimer of responsibility. The Bone@BC app is approved by the Danish Breast Cancer Patients Society and the unified Danish eHealth Portal [68]. Licenses are obtained for included patient-reported questionnaires that require a license.

## Results

The enrollment for the stage 2 medical audit of patients started in November 2022, and lasted until February 2023; a total of 23 patients have been included. The enrollment for the stage 2 semistructured patient interview is ongoing, and a total of 19 patients have now been enrolled.

The scientific findings derived from the study, regardless of being positive, negative, or inconclusive, will be documented in original manuscripts and submitted for publication in peer-reviewed international journals specializing in the relevant field (with authorship defined by the International Committee of Medical Journal Editors criteria) [69]. The results will furthermore be disseminated at relevant scientific conferences and professional meetings as oral presentations, as well as in poster forms. The Vancouver recommendations [69] will be followed in all publications based on the study.

## Discussion

### Principal Findings

This study is, to our knowledge, the first to develop an ePRO platform specific to PRO for patients with postmenopausal nonmetastatic ER+ BC in maintenance therapy with AIs with an endocrinology aspect. Furthermore, to our knowledge, there are no previous studies regarding the development of an electronic app with a user-friendly PCI prompt list. The BELIEVE@BC study will contribute knowledge about how the use of an app for women with BC can be a helpful symptom management tool in their everyday lives. The BELIEVE@BC study will also contribute knowledge about whether the use of an app can be an important communication tool during consultations with HCPs. The use of ePROs offers HCPs an improved understanding of patients’ symptoms during the intervals between their hospital visits.

### Comparison With Previous Work

#### Overview

Due to improvements in diagnostics and treatments, the 5-year survival rate for patients with BC is 90% after the initial diagnosis [70]. During treatments, patients typically have consultations weekly and then gradually reduce to annual visits [71]. During this transition from hospital-based care to health self-management, the patients with BC are encouraged to exercise because of accumulating evidence for the efficacy of exercise training in cancer survivorship [6,71] and, in the majority of cases, adherence to endocrine treatments to reduce the risk of BC recurrence [9]. There is accumulating evidence that many patients with BC (and other patients with cancer) find this transition physically and mentally difficult due to reduced interaction with psychological support from HCP [5-8]. Additionally, ongoing late side effects, for example, pain, fatigue, and loss of appetite [9], and less support from surroundings, given that their surroundings consider them to be healthy [9], are important factors. The late side effects may develop months or even years afterward, and the patients with BC are therefore, in their everyday lives, troubled with being alone with their burdens. A systematic review [72] included 42 studies of self-management education for patients with cancer. Hereof, 16 studies concerning BC suggest that self-management interventions may reduce symptoms of fatigue, pain, depression, anxiety, and emotional distress and increase HRQoL [72].

By equipping patients with the necessary skills, confidence, and knowledge to self-manage their health, they gain increased autonomy and control over their well-being. This empowerment fosters healthy behaviors and encourages proactive measures to prevent long-term illnesses. In many cases, individuals are capable of managing minor illnesses on their own, leading to a decreased reliance on professional assistance. This enables HCPs to allocate their resources and attention toward providing care for patients at higher risk, particularly those with coexisting medical conditions or comorbidities. Around 1 in 5 visits to the general practitioners are made for social needs such as isolation, management, low mood, and anxiety [73]. A systematic review from 2019 [18] found that mHealth apps with interventions focusing on BC survivorship showed a positive effect. By promoting weight loss, improving HRQoL, and decreasing stress. They find that future research is needed to explore the impact of mHealth apps on patients with BC undergoing maintenance therapy [18]. The knowledge of the use of mobile apps for monitoring patients with BC during maintenance therapy is still limited.

#### Timeline

The study will be conducted over a duration of 3 years, encompassing well-defined stages. The planning of the study appears to be realistic and feasible within the specified timeline. Stage 4 is deemed feasible, and the deliverables are realistic given that the Bone@BC app has already been implemented in a proof-of-concept version.

### Limitations

One of the limitations of this study is that the EORTC Library was chosen for selecting the ePROs in the app. Despite an overlap of primary treatment symptoms from chemotherapy and maintenance therapy with AI, more specific ePROs measurements could have been included in the app. Furthermore, it is important to note that the app primarily addresses symptoms associated with maintenance therapy. However, it may be worth considering the development of a future app that encompasses symptoms related to both primary and maintenance therapies.

### Conclusions

Carefully developed with the involvement of patients and systematically validated, the Bone@BC app may have the potential to be a tool for optimal symptom management for patients with postmenopausal nonmetastatic ER+ BC in maintenance therapy. This protocol outlines the BELIEVE@BC study, which seeks to enhance the care and comprehension of the needs and symptom burden experienced by patients with postmenopausal early BC during maintenance therapy. The patient-friendly version of the Bone@BC app may help to increase patients’ self-efficacy. Increased self-efficacy can lead to improved confidence and engagement in their health care decisions and actions.

## Abbreviations

AI: aromatase inhibitor
BC: breast cancer
CONSORT: Consolidated Standards of Reporting Trials
EORTC: European Organization for Research and Treatment of Cancer
EORTC QLQ-C30: European Organization for Research and Treatment of Cancer Quality of Life C30
ePRO: electronic patient-reported outcome
ER+: estrogen receptor–positive
HADS: Hospital Anxiety and Depression Scale
HCP: health care professional
heiQ: Health Education Impact Questionnaire
HRQoL: health-related quality of life
mHealth: mobile health
MVP: minimum variable product
PCI: Patient Concerns Inventory list
PRISMA-P: Preferred Reporting Items for Systematic Review and Meta-Analysis for Protocols
PRO: patient-reported outcome
SES6G: Self-Efficacy for Managing Chronic Disease 6-Item Scale
SGPALS: Saltin-Grimby Physical Activity Level Scale
SUS-DK: System Usability Scale–Danish version

## Data Availability

Data sharing is not applicable to this article as no data sets were generated or analyzed during this study.
